# Taking the Papers

**Published:** 1861-12

**Authors:** 


// HALL'S JOURNAL OF HEALTH.

Our Legitimate Scope is almost boundless: for whatever begets pleasurable and harmless feelings, promotes Health; and whatever induces disagreeable sensations, engenders Disease.

WE AIM TO SHOW HOW DISEASE MAY BE AVOIDED, AND THAT IT IS BEST, WHEN SICKNESS COMES, TO TAKE NO MEDICINE WITHOUT CONSULTING A PHYSICIAN.

Vol. VIII.] DECEMBER, 1861. [No. 12.

### TAKING THE PAPERS.

IT would save trouble and annoyance, if all who read newspapers and magazines would arrange to have their subscriptions end with December of each year; then they could not "forget" that their subscription had "run out." And let all remember that there is no season of the year wherein publishers most need what is due them, and most need the price of renewals, than the month including the Christmas holidays; for then it is that their hard-working employés want every cent due them to be paid, in order "to make merry" with their families and friends. If they are not thus paid, their holiday-time is clouded, and that of their wives and children. Hence no person from the country can imagine the aggregate of gladness produced, or the sum-total of disquietude and unhappiness caused by the neglect to send the one, two, three, or more dollars owing to publishers, from individuals scattered here and there all over the land. It may be said that almost every dollar sent to publishers between this and the first day of January, will cause a smile of pleasure or a throb of joy to those, or their children, who help to set the type, or print the paper, or fold and stitch the very magazine which you, reader, will be reading and enjoying around your Christmas-fire; to say nothing of that quiet satisfaction which never fails to well up in the bosom of every honest man or woman, boy or girl, on the payment of an honest debt.

We recommend to our readers the policy, wisdom, and justice of subscribing to the paper, of the class they wish to patronize, which is published nearest their dwelling, even if you are not altogether pleased with it. You thus aid, as it were, in improving your own property; and the better any paper of good general principles is supported, the better it becomes, and the more wholesome the influence it exercises in the community amid which it is issued.

Each denomination of Christians should patronize the paper of their faith, in their own State, in preference to any religious paper elsewhere, although many times cheaper, larger, and better. Then, if you have more money to spare, take a paper published in one of the larger cities.

He only is consistent with his profession who is "firmly persuaded in his own mind" that his own sect, his own religious faith, is the nearest right of any other; hence the "liberality" which leads a man to neglect the paper of his own church, and subscribe for that advocating another "faith," is the liberality of ignorance, indifference, or hypocrisy. Such a man is worse than nobody in any church, and ought to be "spewed out" of his society as a mere "cumberer," that some more worthy may occupy his place.

Inquiries are made of us, from time to time, as to what paper or magazine we would recommend. We consider *The American Agriculturist*, New-York, monthly, one dollar a year — German edition the same — to be, by all odds, the best, cheapest, and most ably edited of any agricultural magazine in the United States. Of the agricultural newspapers, the *New-England Farmer*, Boston, two dollars a year, and that time-honored and general favorite, *The Country Gentleman*, Albany, N. Y., same price, are at the head of their class, and have such a start that no doubt they will continue their supremacy.

For the region south of New-York State, *The American Farmer*, monthly, two dollars a year, Baltimore, Md. For the north-west, we commend *The Prairie Farmer*, Chicago, and *The Farmer*, at Detroit, Michigan. Besides, these last two contain general reading well adapted to all farmers' families; the articles being uniformly selected with taste and judgment.

Of the very best Baptist newspapers in the nation are *The Examiner*, of New-York, and *The Christian Watchman and Reflector*, Boston. Either of these papers can be safely and instructively patronized by any orthodox family. *The Examiner*

seems always to feel itself to be a gentleman, as well as a Christian, while *The Watchman*, true to its name, is more faithfully on the look-out against "heresy, infidelity, and schism," than any religious rewspaper we receive; and what is more, on the look-out for these things in places where they might not be suspicioned to exist, and would otherwise be allowed to lurk, and corrupt and poison to a most hurtful extent. It has found bad doctrine and pernicious sentiments in weeklies, monthlies, and quarterlies, and in bound volumes too, which so keen an eye as the veteran *New-York Observer* has allowed oversightedly to pass muster; and for its fidelity, able as well as fearless, all orthodoxy owes patronage to the *Christian Watchman and Reflector* of Boston, Mass.

No good Old School Presbyterian in all the Mississippi Valley, will send a single dollar east for a religious newspaper, until he has first subscribed and paid for *The Presbyterian Herald*, of Louisville, Kentucky, a paper we used to write for when we were a medical student, so many years ago — we do not like to state the figures of the same, beyond that it is over a quarter of a century— and some of these same pieces we have seen within a year come up to the surface again, with a lost paternity. The editorials of the *Herald* have, within the last year, been more frequently copied into the secular as well as religious papers of various denominations, at least as far as our exchanges are concerned, than those of almost any other paper. This fact should show Dr. Hill that his pen is wanted; and that while he has the ability he ought to keep it going, for none can know how soon the moment may come with the call to "go up higher!" *The Presbyterian Banner*, of the olden faith, well merits the patronage of its Church, at Pittsburgh, and many a league round about. As for the other paper of the same sentiments, or more so — between the *Banner* and the *Herald* — namely the *Presbyter*, of Cincinnati, we can't say that we like it so well. It is so unbending, so John Knoxy, that it leans over on the other side. Perhaps, after all, these fellows of ice and steel, straight-lined and right-angled, have a useful nichc to fill in these accommodating and time-serving ages, when the loaves and the fishes are "gods." We rather think, on the whole, that if Paul had been an editor, he would have been a "Presbyter" too, at least on a good number of points theologic. Of *The*

*Presbyterian*, the father of the faithful, and of all Presbyterian paperdom, we need say nothing ; it has been a faithful servant of that Church, and a powerful champion for its rights and its purity; and no good Presbyterian ought to take any other religious paper out of his own church, to the exclusion of *The Presbyterian*, of Philadelphia. A baby in years is the *Standard*, of the Old School, at the same place and price. There was no use in starting it, in the first place. There are altogether too many religious newspapers for the real good of the churches. Orthodoxy would be largely the gainer, if four out of five of the whole of them were abolished beyond the power of resurrection. One well-sustained and ably-edited paper, as the organ of any sect, is worth a regiment of puling, wishy-washy things, only born to live in death-struggles, and finally go out in debt, dishonor, and disgrace. But we must say of the *Standard*, as many a mother has said lovingly of her new-born babe: "I didn't want you to come, but now you are here, bless your dear little heart, I love you all to pieces." *The Standard* has, up to this time, been edited with a steady ability, judgment, and discrimination, and has thus shown itself worthy of a liberally-sustained patronage, which we hope it will secure.

*The American Presbyterian*, of Philadelphia, and the *Central Christian Herald*, of Cincinnati, are the organs of the New School Presbyterian Church. They both have been faithful in their day and generation, and sphere, and deserve that every subscriber should not only pay every cent owing before Christmas, but should send up another new name, for the "good of the cause." He who befriends these papers thus, in these "troublous times," is a true friend. Those who do nothing, even scarcely paying their balances, ought to be placed within leg-distance of that obese individual named in Deuteronomy, 32:15.

There are two papers published at Dayton, Ohio. We always, *always* open them with interest, because they seem to be edited with ability, conscientiousness, and discrimination. We have been reading them for years, and when we don't scissor them up totally, we send them to our country cousins all about; they are too good to be destroyed. A dowager Presbyterian lady, who has had the run of our exchanges, and is just going into her new mansion on Murray Hill, said to us yesterday: "I

must take the *New-York Observer*, of course, but I am going to order the *Western Missionary*, of Dayton, Ohio." It is only a dollar a year, and its neighbor, *The Religious Telescope*, somewhat larger, (either is large enough,) is one dollar and fifty cents. We really do not know of what denomination or society these papers are, but they are Christ's, and surely that is enough. They are on his side *all the time*, and that is saying a good deal. In fact it is much more than can be said of quite a number of so-called religious newspapers, too many of which are given to gouging each other's eyes, faith, and character. Wonder if it's because the "Old Boy" is among them at such times, a gymnasticizing them, giving them Zouave lessons in theological fisticuffs, somersets, and the like?

The New-York *Evangelist* has been so long known, and so many of its subscribers are readers of our own journal, that we need only remind them that the very least each one of them ought to do, after settling arrearages and paying up for 1862, should be to go around among friends, and get up two or three names a piece, as a deserved and substantial testimonial of your appreciation of what that paper has ably and faithfully done.

*The Christian Intelligencer* of New-York is the organ of the Reformed Dutch Church. It has been so conducted for many years, that it has not only gained the appreciation of its own people, but has secured the respect and confidence of the religious press throughout the land; and any Reformed Dutch family which does not take and punctually pay for this old friend of the true and pure doctrines of the Bible, is not doing its duty, and ought to be ashamed of itself. There is one paper whose name has been familiar to us from early childhood; it was the first religious newspaper we ever saw—the first we ever read. It was a familiar sight on grandmother's knee, a long time ago, away out yonder in the wild woods of the West, when merchants used to pass by our door, coming "East" for goods; driving "pack-horses" before them laden with Spanish dollars, which had made their long and weary way from Mexico to "Nu-orléenes," as it was then called, up the Mississippi, or *via* Santa Fé and St. Louis. It was then a poor, little, coarse, yellow quarto, but always contained so many good things, so much missionary news, so much pure, Christian counsel, admonition, and encouragement, that it was read and lent, and brought home and read again, then saved and bound, and cherished, long years after, as a treasure and a friend. This paper "still lives" by its old and time-honored name of *Boston Recorder;* and if its subscription-list of long time ago could be found, the name of Hannah Pyke, with "nary red" ever found against it, would be seen running through many successive years, until the Master called her

"To be an angel too."

There are two other papers which we have not by any means forgotten: the *New-York Chronicle*, Baptist, and *The Banner of the Cross*, Philadelphia, Episcopalian. We trust they both receive, as they well deserve, a liberal patronage. The *Banner* comes about as near being what a religious newspaper ought to be, as most that come to our table. It is about half the size of a common newspaper, and in this is one foundation of its merit. The needed variety requires pith and condensation; excluding altogether, and remorselessly, that immense mass of hybrid, mongrel matter, neither "civil" nor religious; a compound that coälesces and makes neither the one nor the other, and whose effect on the mind is very "evil." Just look at the long columns of "foreign correspondence," hosannas of patent-medicines, and of the very "patent" men who lie so vigorously about their merits. Don't seem to us that there is much religious reading in these things—in "sooth ing syrup" made out of opium, and *can not be made* of any thing else, as every intelligent, honest druggist will confess; still less in Bourbon whisky diluted with cream; and bitters, always made of alcohol, it being the agent that *must* be employed to extract the bitter principle. And yet these same papers would be horrified at being asked to insert an advertisement as to where opium would be sold, to cure every pain, and where a glass of egg-nog, brandy-toddy, or sangaree could be always had at a "moment's notice," "warranted," in every instance, to make one feel better, with the advantage of there being no lie in the last assertion, at least according to our experience lang syne. We vote, then, that all the religious newspapers reduce their size to that of the *True Union* of Baltimore, and, like it, be always readable, always awake, always on the side of a true and earnest piety. And Sir Oracle saith further:

Keep up your prices, and never trust another dollar for a single hour. If the churches do not sustain you, let theirs be the responsibility: you have done your duty; and there are other fields in which the same ability, the sameindustry, and the same mental power will return a much more abundant pecuniary reward.

There are two papers which we can not say are good, better, or best, for they are alone in their glory; and, consequently, peerless in their sphere. Both of them are so neat in their exterior, that the very fact of a man's having one of them in his hand is *prima facie* evidence that he is a person of refinement, or of superior education in his line. *The Home Journal* and *The Scientific American*, issued weekly in New-York, at two dollars a year. These papers are so well adapted to the spheres they were intended to fill, and so completely fill them, that they have no rivals; there is no room for rivalry, and it would be no use to attempt it. There is but one Morris and Willis on this or any other continent, and Munn & Co. will, as they have done, stand alone for many a long year; and in ability, too, as well as in prosperity. Every admirer of the good John Wesley — the fearless and indefatigable worker — who lays any claim to culture, elevation, and breadth of view, will find *The Methodist* of New-York, two dollars a year, the most ably edited weekly in that large denomination, whether in this country or *any where else.* So much for the batch of our OLD newspaper exchanges *received last Saturday*, except two; and yet, with "one consent," all the names mentioned, except the *Home* and the *Scientific*, who would not be taken as *very* pious, by any crowd, had "rather not" have them mentioned. But we beg leave to mention them without meaning to "offend one of" the "little ones." If any of our readers should be offended, and pout a little, and determine not to take our JOURNAL any more, we can only say, pout on until you are tired, and get a little more sense. We say this defiantly, because we know you can't well do without us, and like any other infant, you will come around after a while, and do the very thing you resolved you wouldn't do. The first is a Universalist paper at Boston, Mass., two dollars a year, called *The Trumpet.* We do not believe that Calvin, or John Knox, or "Bob Breckinridge," their successor in a direct line, and of the pure blood unadulterated, undiluted, and undeteriorated, even under the influence of a dyspeptic dinner, could find it in their hearts to erase one line in a month from its fourth page of general reading matter; and there is not a family paper in the land that would not be improved, in our opinion, by copying weekly this same fourth page of reading matter. The first page is doctrinal. The inner pages are devoted to advertisements and their home matters. The other paper is *The Catholic Herald* of Philadelphia. As to its peculiar tenets, we say nothing; but we have a high respect for the talents and industry of the man who can, every week, sift out so much general intelligence for his readers, in connection with a large assortment of useful and unexceptionable general reading. Notwithstanding we have taken up so much space for the benefit of our *confrères*, we must take up still more; for the reading of families is a moral medicine of great value, and this JOURNAL has long since assumed the province of medicating the mind and morals, as well as the brain and body. The fashions of the times are such, that every family having any pretension to intelligence, respectability, or position, feels a desire not only to have a daily or weekly newspaper, but also to take some magazine. To the higher classes, who have both means and cultivation, we say, with the utmost confidence, that ten dollars can not be spent to greater advantage in this direction, than in the procurement of the republications of Leonard Scott & Co., of 79 Fulton street, New-York, to wit:

| | |
|---|---|
| Blackwood's Magazine, monthly, | $2.00 |
| London Quarterly, (Conservative,) | 3.00 |
| Edinburgh Review, (Whig,) | 3.00 |
| North British Review, (Free Church,) | 3.00 |
| Westminster Review, (Liberal,) | 3.00 |

Or the whole for ten dollars a year, delivered free of postage in all the principal cities and towns. These are written for by the best scholars and strongest minds in Great Britain; and the educated have a feast in reading them, although there may not be coïncidence of opinion at all times. The *Westminster Review* is so often infidel in its sentiments, that a good many of its numbers would serve a better purpose, as to society's best good, by being thrown into the fire.

For monthly reading of a religious cast, always safe, *The*

*Home Monthly*, Boston, two dollars a year, is the very best; Rev. William M. Thayer, chief editor. Those who want lighter reading, that which is not specially religious, will find it in T. S. Arthur's *Home Magazine*, Philadelphia, two dollars a year. For purity of style and subject, Mr. Arthur has made for himself an enviable reputation. He never printed a line that any one, however religious or refined, need hesitate to read at any family fireside, of "wife, children, and friends." *Godey's Ladies' Book* maintains its popularity with the multitudes who have patronized it for so many years, and is so firmly established in their partialities, that they will take care of it "any how."

The gentle Woodworth is no more, but the boys and girls will, for his sake as well as their own, continue to "take" *Merry's Museum* until they are boys and girls no more.

There is no mother in the land who could possibly fail to derive most important assistance, in the discharge of her responsible and momentous duties, by taking some magazine designed to give hints, aids, and instruction as to the moral training of the family. One of the very oldest and best of this class is the *Mother's Journal*, New-York, one dollar a year, edited so long, so industriously, so conscientiously, and well, by Mrs. Caroline E. Hiscox. It is quite a mistake to suppose, that with our schoolboy days we are to cease learning orthography, etymology, syntax, and prosody. Language is changing; words are changing; grammatical rules and constructions are changing; and we ourselves must change with them, or to a dead-fire certainty we will become old fogies before we are forty. It is a very pleasant sight to see gray-headed people up to the times, instead of being put in the background, as is too often done. We were thinking last night, as the fire beamed and burned so brightly and cheerily in our low-down grate, and the children all quite as busy as any minister of state, although ten thousand times happier, as dimpled cheeks, snatches of song, and the loud laugh very conclusively proved—we repeat, while we contemplated the happy scene, and remembered that the "gray-hair" who would have enjoyed it too, to the full, as much as we did, had taken up her returnless journey since a year ago, that old age added to the beauty and happiness of the family-fireside; and we were on the point of laying a plan by which we could get some nice, cheery, kind-hearted old person to supply the vacancy, when we remembered sadly, that but too soon there would be two others to fill the niche without going from under our own roof. But "to return to the sheep." There is a class of monthly publications called "Teachers," designed primarily for those who teach school, to impart every variety of information calculated to aid in governing and educating children. These monthlies abound in valuable and suggestive hints, which can well be appropriated by parents, and would be of inestimable service to them, in enabling them the better to manage their own children; to lead out their capabilities; to mold their characters; to control their passions and propensities; and to cherish the germs of goodness in the heart, of excellence in the character, of power in the mind; for much of what these publications contain, is the interchange of teachers' experience, observation, and practice. Another every-day practical good to be derived from these issues is, that important suggestions and rules are laid down as to words and phrases; as to pronunciation and grammar, so that there is not a household in the land that would not find amusement and profit in reading these "Teachers" in the family circle, and in following them up in polite, kindly, and courteous criticisms, as to each other's *lapsus linguæ*, and delinquencies of speech, pronunciation, and grammar. The "Teachers" are only a dollar a year, and we are fully persuaded that a dollar could not be spent to greater advantage, in the direction of the improvement and elevation of a family in these regards. Send, then, a dollar, "on receipt of this," to *The Teacher*, at Albany, N. Y., or Boston, Mass., or Portland, Me., or *Home and School Journal*, Chicago, Ill., or *Journal of Education*, Ann Arbor, Mich.

